# PERK/eIF2α signaling inhibits HIF-induced gene expression during the unfolded protein response via YB1-dependent regulation of HIF1α translation

**DOI:** 10.1093/nar/gky127

**Published:** 2018-02-26

**Authors:** Iglika G Ivanova, Catherine V Park, Adrian I Yemm, Niall S Kenneth

**Affiliations:** Institute for Cell and Molecular Biosciences, Faculty of Medical Sciences, Newcastle University, Newcastle upon Tyne NE2 4HH, UK

## Abstract

HIF1α (hypoxia inducible factor 1α) is the central regulator of the cellular response to low oxygen and its activity is deregulated in multiple human pathologies. Consequently, given the importance of HIF signaling in disease, there is considerable interest in developing strategies to modulate HIF1α activity and down-stream signaling events. In the present study we find that under hypoxic conditions, activation of the PERK branch of the unfolded protein response (UPR) can suppress the levels and activity of HIF1α by preventing efficient HIF1α translation. Activation of PERK inhibits de novo HIF1α protein synthesis by preventing the RNA-binding protein, YB-1, from interacting with the HIF1α mRNA 5′UTR. Our data indicate that activation of the UPR can sensitise tumor cells to hypoxic stress, indicating that chemical activation of the UPR could be a strategy to target hypoxic malignant cancer cells.

## INTRODUCTION

Cellular hypoxia can occur as a consequence of low atmospheric oxygen or locally in tissues due to inflammation, ischemia, injury or poor vascularisation (1). At the cellular level, hypoxia is characterised by a switch in energy metabolism coupled with a rapid change in the transcriptional program, primarily mediated by the hypoxia inducible factor (HIF) family of transcription factors (1,2). Activation of HIF promotes the expression of specific target genes that play critical roles in the adaptive response to hypoxia and the restoration of cellular homeostasis (2).

HIF1 is a ubiquitously expressed heterodimeric transcription factor, composed of an oxygen labile HIF1α subunit and a constitutively expressed HIF1β subunit (2). HIF1α and HIF1β are essential for development as both HIF1α and HIF1β knockout mice die in utero between 9.5 and 10.5 days of gestation, largely due to defects in embryonic vascularisation (3–5). HIF1α stability is primarily regulated through the action of several proline hydroxylases (PHDs), which act to modify proline residues in the oxygen-dependent degradation (ODD) domain of HIF1α (6). Hydroxylated HIF1α is recognised by the von-Hippel Lindau (VHL) E3-ubiquitin ligase, which promotes the ubuiquitination and subsequent degradation of HIF1α by the 26S proteasome (7). As a consequence, the half-life of the HIF1α protein is <5 min in normal conditions, resulting in the HIF1α protein being virtually undetectable in adequately oxygenated cells and tissues (8,9). In hypoxic cells PHD enzymes are inhibited resulting in rapid HIF1α accumulation, this allows HIF1α to dimerise with HIF1β to promote the expression of HIF target genes (1,2).

Although HIF1α levels are primarily regulated by proteasomal degradation alternative mechanisms exist to modulate HIF activity such as transcriptional regulation of HIF genes or post-translational modification of HIF subunits (10). Control of HIF1α biogenesis through regulation of protein translation is also emerging as an important mechanism for regulating HIF in hypoxic cells. In fact, HIF1α protein biogenesis is responsible for 40–50% of the increased levels of HIF1α protein in response to hypoxic stress (11,12). HIF1α has both 5′ and 3′ UTRs that can regulate its translation; with the 5′ UTR containing an internal ribosome entry site that can upregulate HIF1α translation, and the 3′ UTR mainly responsible for controlling mRNA stability (13). 5′-UTR-dependent upregulation of HIF1α translation is observed in metastatic cell lines, indicating that this mechanism of HIF1α elevation may be critical for the malignant phenotype (13).

In actively growing eukaryotic cells, protein translation accounts for ∼75% of the total energy expenditure of a cell (14). During severe hypoxia/anoxia (<0.2% O_2_), cellular energy consumption is limited and global protein synthesis is inhibited through activation of the unfolded protein response (UPR) (15). The UPR is a highly conserved pathway that allows cells to effectively manage cellular stress triggered by chemical and environmental factors (16). Central to the UPR is the PKR-like ER kinase (PERK)-dependent phosphorylation of eukaryotic initiation factor 2α (eIF2α) which represses global translation while promoting the preferential translation of mRNA that encode stress-responsive factors to restore cellular homeostasis (16,17). During severe hypoxia/anoxia the UPR and hypoxia response pathways interact to potentiate the expression of HIF target genes (18). However, inhibition of the PHD enzymes and stabilisation of HIF1α occurs at relatively moderate levels of hypoxia (<2%), which is not sufficient to activate the UPR (19).

In this present study, we examined the consequences of activating the UPR in conditions of moderate hypoxia to investigate if this could potentiate the HIF-dependent hypoxic response. Surprisingly, we find that chemical activation of the UPR during moderate hypoxia impairs HIF1α stabilisation and results in the down regulation of hypoxia-induced HIF1 activity. Our data indicate that activation of the UPR in low oxygen severely reduces HIF1 activity by blocking HIF1α mRNA translation in a PERK-dependent manner. Activation of the UPR reduces the interaction between the RNA binding protein, YB-1, and the 5′-UTR of the HIF1α mRNA, thus preventing its efficient translation. Chemical inhibition of PERK rescues the HIF1α defect and the levels of HIF1 activity in hypoxic cells treated with UPR agonists. Impairment of the HIF pathway by UPR activation results in a reduction of cell viability in low oxygen, indicating that targeting the UPR may be a strategy to target hypoxic malignant cells.

## MATERIALS AND METHODS

### Cell lines

PC-3 cells were grown in RPMI with 25mM HEPES, supplemented with 10% FBS and l-glutamine. U2OS, MCF7 and COV-434 cells were gown in DMEM supplemented with 10% FBS, l-gluatamine, and penicillin streptomycin. U2OS HRE luciferase and U2OS NF-κB luciferase cells have been previously described (20).

### Treatments

Cells were incubated at 1% O_2_ in an *in vivo* 400 hypoxia work station (Ruskin, UK). Cells were lysed for protein extracts, and RNA extraction in the chamber to avoid re-oxygenation.

Thaspsigargin (Enzo), tunicamycin (Calbiochem), DMOG (Calbiochem), GSK2606414 (Calbiochem), MG132 (Sigma), lactacyctin (Calbiochem), ionomycin (Sigma) were dissolved in DMSO and added to cells at the concentrations indicated in the figure legends. DTT (Sigma) and EGTA (Sigma) were prepared in ultrapure water.

### Cell lysis and immunoblotting

Cells were lysed in 8 M urea lysis buffer and immunoblotted as described (21). Antibodies used were HIF1α (Clone 241809, R&D systems), HIF2α (#7096, Cell Signaling Technologies), phospho- eIF2α (Ser 51) (#9721, Cell Signaling Technologies), eIF2α (#5324, Cell Signaling Technologies), PERK (#3192, Cell Signaling Technologies), YB-1 (A303–231A, Bethyl), β-tubulin (#2146, Cell Signaling Technologies), VHL (#68547, Cell Signaling Technologies), β-actin (AC-74, Sigma).

### Luciferase assays

Lysates for luciferase assay were prepared in 1× passive lysis buffer (Promega), 100 μl per well of a 24-well plate. 10 μl of lysate was incubated with 50 μl luciferase reagent (Promega) and measured for 10 s using (Lumat LB9507, EG&G Berthold). Graphs are represent raw RLUs readings from three independent experiments.

### Polysome profiling

PC-3 cells were grown to 80% confluency and incubated in 100 mg/ml cycloheximide for 3min and resuspended in hypotonic polysome extraction buffer (5 mM Tris [pH 7.5], 2.5 mM MgCl_2_, 1.5 mM KCl, 1% Triton X-100, 100 mg/ml cycloheximide, 100 U/ml RNasin). Cell were lysed through the addition of Triton X-100 (0.5%) and sodium deoxycholate (0.5%) to solubilise the cytosolic and endoplasmic reticulum-associated ribosomes. Extracts were normalised by OD 260 nm and layered onto 10 ml of 10–50% sucrose steps and centrifuged at 222 228 × g (36 000 rpm) for 2 h at 4°C using SW41Ti rotor. The sucrose steps were fractionated into twelve 0.75 ml fractions. Absorbance at OD_254 nm_ and visualisation by RNA agarose electrophoresis following Trizol (Invitrogen) purification was used to determine the monosomal and polysomal fractions.

### RNA immunoprecipitation

To probe for direct interactions between YB-1 protein and HIF1α transcripts PC-3 were resuspended in hypotonic polysome extraction buffer and lysed through the addition of Triton X-100 (0.5%) and sodium deoxycholate (0.5%). Clarified lysates were incubated with 1 ug of YB-1 antibody and antibody/protein/RNA complexes were isolated by using 20 μl packed volume protein A sepharose beads. Beads were washed with polysome extraction buffer and RNA isolated using the PeqGold RNA isolation kit. cDNA was prepared using Qiagen Quantanova cDNA synthesis kit.

### Quantitative reverse transcription-PCR

Quantitative PCR data was generated on a Rotor-Gene Q (Qiagen) using the following experimental settings: hold 50°C for 3 min; hold 95°C 10 min; cycling (95°C for 30 s; 58°C for 30 s; 72°C for 30 s with fluorescence measurement for 45 cycles). All values were normalised to 18S rRNA or RPL13A levels using the Pfaffl method as indicated. Primers sequences: HIF1α For- 5′-CATAAAGTCTGCAACATGGAAGGT-3′, HIF1α Rev 5′-ATTTGATGGGTGAGGAATGGGTT-3′; 18S rRNA For 5′-GTAACCCGTTGAACCCCATT-3′, 18S rRNA Rev 5′- CCATCCAATCGGTAGTAGCG- 3′; RPL13A sense 5′-CCT GGA GGA GAA GAG GAA AGA GA -3′, antisense 5′-TTG AGG ACC TCT GTG TAT TTG TCA A-3′; BNIP3 sense 5′-GCC CAC CTC GCT CGC AGA CAC-3′; GLUT1 sense 5′-CTG GCA TCA ACG CTG TCT TC-3′, antisense 5′-GCC TAT GAG GTG CAG GGT C-3′; PDK1 sense 5′-AGT TCA TGT CAC GCT GGG TA-3′, antisense 5′-CAG CTT CAG GTC TCC TTG GA-3′.

### Cell viability assays

Cell viability was measured using Prestoblue assay (Invitrogen) and performed according to the manufacturer's protocol. Briefly, PC-3 cells were seeded at a density of 5000 cells/well. Cells were pre-treated with inhibitors for 30 min before incubation at 1% O_2_ or 21% O_2_ for 24 h. The absorbance was recorded at 570 nm after 30 min incubation of cells with Presto Blue reagent. The cell viability was expressed as a percentage relative to untreated controls.

## RESULTS

### ER stress suppresses HIF levels and activity in moderate hypoxia

**Figure 1.**
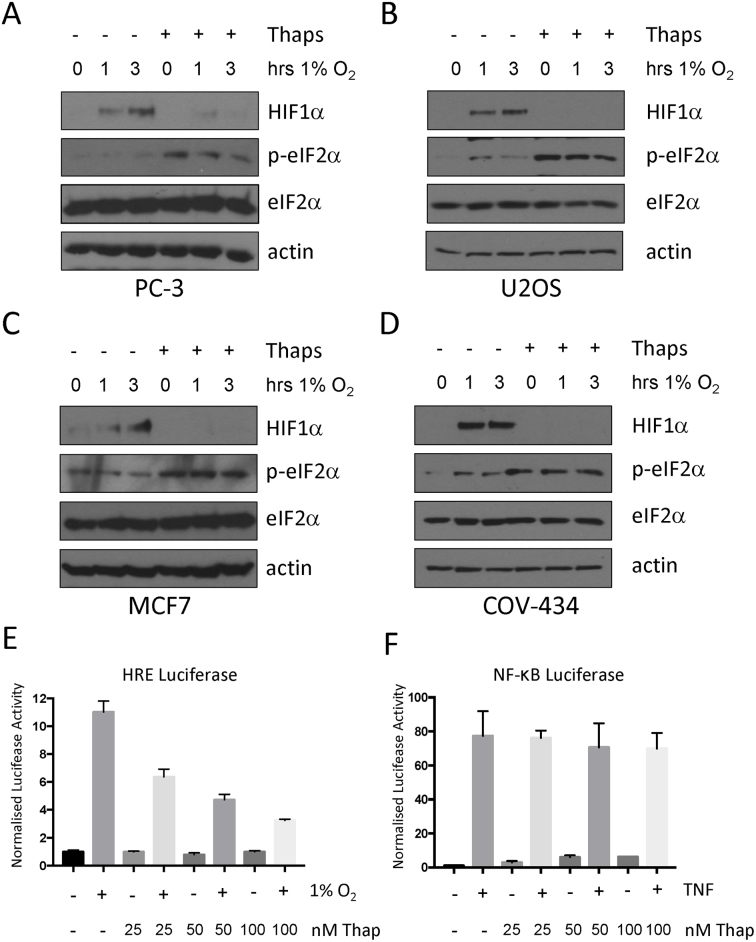
Reduction of HIF1α accumulation and activity in thapsigargin treated cells. (**A**) PC-3, (**B**) U2OS, (**C**) MCF7 or (**D**) COV-434 cells were treated with 50nM thapsigargin as indicated and exposed to 1% O_2_ for the indicated times. Whole-cell lysates (WCLs) prepared from these cells were subjected to immunoblot analysis to assess expression levels of the indicated proteins. (**E**) U2OS cells stably expressing luciferase driven from the HRE promoter element (HRE-Luc) were treated with the indicated concentrations of thapsigargin and exposed to 1% O_2_ for 7 h. (**F**) U2OS cells stably expressing luciferase driven from the NF-κB promoter element (κB-Luc) were treated with the indicated concentrations of thapsigargin and stimulated with TNF for 7 h. Results presented represent the mean plus S.D. of three experiments. Values in E and F are normalised to the respective untreated control.

The endoplasmic reticulum (ER) is responsible for performing multiple functions essential for cellular homeostasis, development, and stress responsiveness (22). Severe hypoxia/anoxia (<0.2% O_2_) induces ER stress and results in potent activation of the UPR (23). However, there are conflicting reports as to whether activation of the UPR during moderate hypoxia contributes positively or negatively to the HIF-dependent transcriptional response (12,24,25). To examine the effect of activating the UPR on the HIF response during moderate hypoxic stress (1% O_2_), PC-3 prostate cancer cells were treated with thapsigargin to induce ER stress (26). Eukaryotic initiation factor 2α (eIF2α) is rapidly phosphorylated in cells treated with thapsigargin, consistent with the UPR being activated (Figure 1A). Moderate hypoxia (1% O_2_) was not sufficient to induce the UPR, as no hypoxia-dependent increase in phospho-eIF2α was observed (Figure 1A). Surprisingly, hypoxia-dependent HIF1α stabilisation was markedly decreased in hypoxic PC-3 cells pretreated with thapsigargin compared to controls (Figure 1A). The effect of thapsigargin on HIF1α stabilisation was tested in MCF7 (breast), COV-434 (ovarian) and U2OS (osteosarcoma) treated with thapsigargin and exposed to hypoxia. In each of the cell lines tested HIF1α stabilisation was impaired in cells in which the UPR is activated indicating that this effect is conserved between cell lines (Figure 1B–D).

HIF activity was then assessed using U2OS cells containing an integrated luciferase reporter construct possessing three copies of the hypoxia-responsive element (HRE) consensus-binding site. A robust activation of luciferase activity was observed in cells exposed to 1% O_2_, which was reduced by treatment with thapsigargin in a dose-dependent manner (Figure 1E). Thapsigargin treatment did not alter TNF-induced NF-κB activation, indicating the effect on HIF activity is specific (Figure 1F).

Previous reports have suggested that modulation of intracellular calcium in hypoxic cells can contribute to HIF-dependent gene expression, both positively and negatively, by modulating its levels and/or its activity (12,24,25). As thapsigargin activates the UPR by significantly altering calcium homeostasis we tested if modulation of intracellular calcium was sufficient to control HIF1α levels. PC-3 cells pre-treated with the calcium ionophore, ionomycin, had similar levels of hypoxia induced HIF1α stabilisation as compared to controls (Supplementary Figure S1A and B). Similarly, chelating excess calcium using EGTA did not significantly alter the levels of HIF1α stabilised by low oxygen (Supplementary Figure S1C). Co-treatment of cells with thapsigargin and EGTA did not alter the thapsigargin-dependent inhibition of HIF1α stabilisation, indicating that ER stress rather than modulation of calcium homeostasis alters hypoxia-induced HIF1α levels (Supplementary Figure S1D).

### Activation of the UPR impairs HIF activity

**Figure 2.**
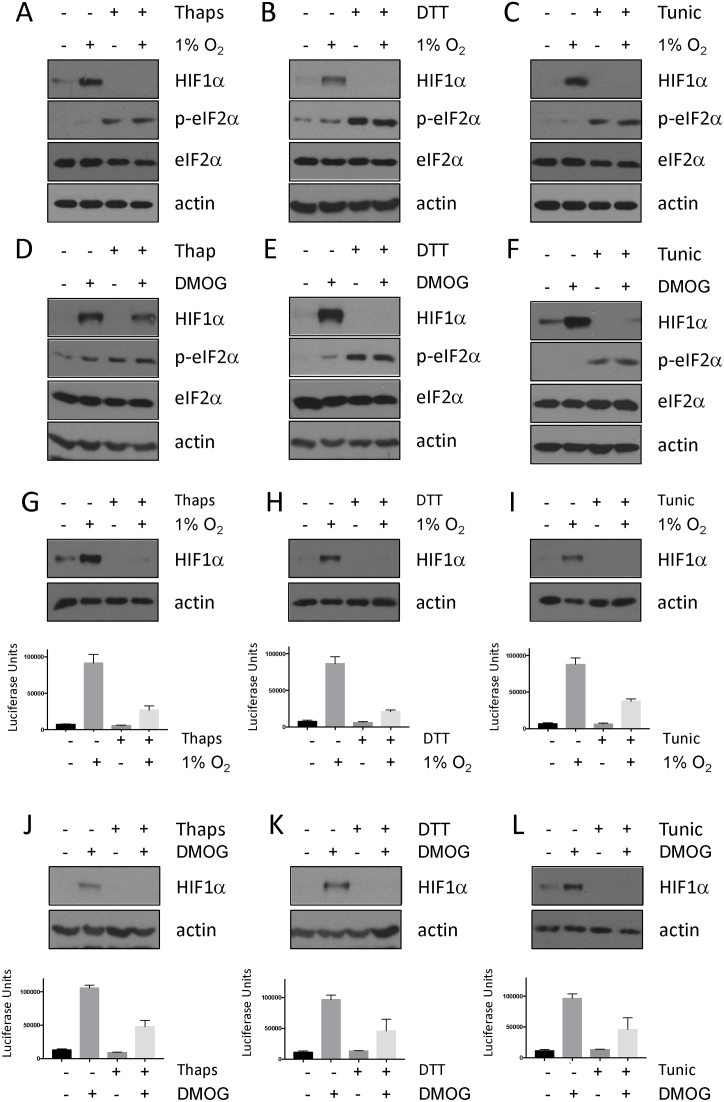
Activation of the UPR reduces the accumulation of HIF1α and HIF1 activity in response to hypoxia or PHD inhibition. PC-3 cells treated with (**A**) 50 nM thapsigargin, (**B**) 2 mM DTT, (**C**) 2.5 μg/ml tunicamycin were subsequently exposed to 1% O_2_ for 3 h. WCLs prepared from these cells were subjected to immunoblot analysis to assess expression levels of the indicated proteins. PC-3 cells treated with (**D**) 50 nM thapsigargin, (**E**) 2 mM DTT, (**F**) 2.5 μg/ml tunicamycin were subsequently treated with 1 mM DMOG for 3 h. WCLs prepared from these cells were subjected to immunoblot analysis to assess expression levels of the indicated proteins. WCLs and luciferase activity was measured from U2OS cells stably expressing HRE-luciferase treated with (**G, J**) 50 nM thapsigargin, (**H, K**) 2mM DTT or (I, L) 2.5 μg/ml tunicamycin and subsequently exposed to 1% O_2_ (G–I) 1mM DMOG (J–L) for 7 h, as indicated. WCLs were resolved by SDS PAGE and analysed by immunoblot using the indicated antibodies. HRE luciferase results are raw luciferase values and represent the mean plus S.D. of three experiments.

To investigate whether alternative activators of the UPR interfere with HIF activity PC-3 cells were treated with diethiothretol (DTT) or tunicamycin. These agents robustly activate the UPR with modes of action distinct from one another, and from thapsigargin (27). DTT and tunicamycin both activate the UPR, as measured by phospho-eIF2α, to a level similar to that seen following thapsigargin treatment (Figure 2A–C). Treatment with both DTT and tunicamycin suppress HIF1α levels following hypoxic stress, consistent with data from thapsigargin treated cells (Figure 2B and C). Stabilisation of HIF1α using the PHD inhibitors DMOG or CoCl_2_ is also sensitive to activation of the UPR, indicating that UPR-dependent decrease in HIF activity is oxygen independent (Figure 2D–F, Supplemental Figure S2). HIF1α levels and activity were also compromised in U2OS HRE-Luc cells pre-treated with UPR activators and exposed to hypoxia or DMOG, consistent with results from PC-3 cells (Figure 2G–L).

### Activation of the UPR does not alter HIF1α protein stability

HIF1α protein levels are primarily controlled by ubiquitin-mediated proteolysis (2). Previous work has indicated that inducing ER stress through modulation of calcium levels can interfere with the ubiquitin-mediated degradation of HIF1α (28). However, treatment with the proteasome inhibitor, MG132, did not significantly increase HIF1α levels in thapsigargin treated cells indicating thapsigargin is not acting to modulate HIF1α protein stability (Supplemental Figure S3A and B). HIF1α could not be stabilised with MG132, or an alternative proteasome inhibitor, lactacystin, in the presence of thapsigargin, suggesting that UPR-dependent modulation of HIF1α is independent of proteasomal degradation (Supplemental Figure S3C and D). HIF1α protein levels can also be modulated by autophagic degradation (29), however lysosome inhibitors failed to rescue the levels of HIF1α in thapsigargin treated PC-3 cells, indicating UPR-dependent control of HIF1α is independent of this signaling pathway (Supplemental Figure S3E and F). Together these data indicate that UPR activation does not modulate HIF1α levels by altering HIF1α protein stability.

### Activation of the UPR reduces HIF1α translation

**Figure 3.**
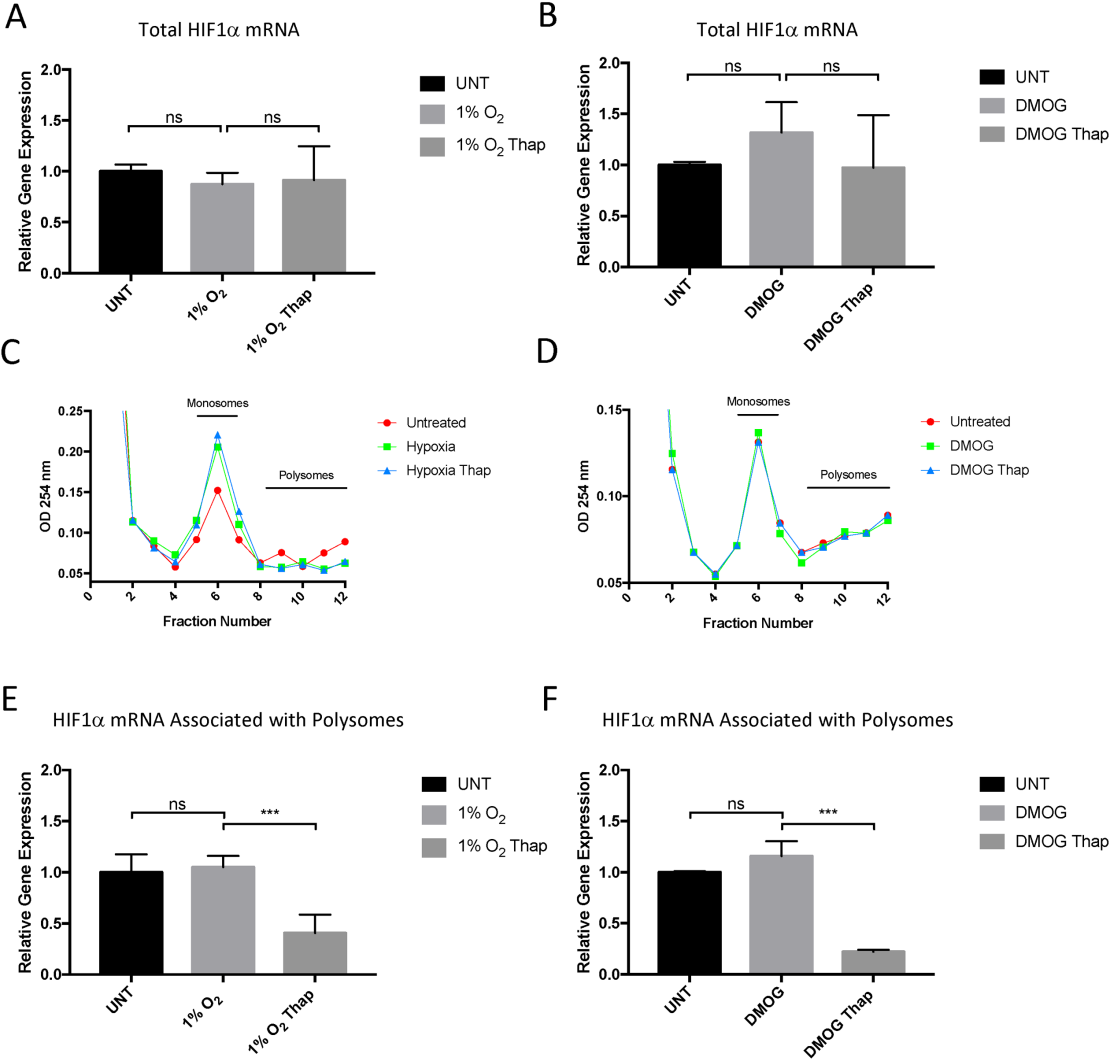
Activation of the UPR suppresses translation of HIF1α mRNA. Quantitative RT–PCR analysis of HIF1α mRNA prepared from PC-3 cells treated with thapsigargin (50 nM) and (**A**) 1% O_2_ or (**B**) DMOG (1 mM) as indicated. Values are normalised to 18S rRNA and fold change calculated from control samples prepared from untreated cells. Representative polysome profiles of PC-3 cells treated with thapsigargin (50 nM) and exposed to (**C**) 1% O_2_ or (**D**) DMOG (1 mM) for 7 h. Fractions containing monosomes and polysomes are indicated on the graph. Quantitative RT-PCR analysis of HIF1α mRNA prepared from polysomal fractions (8–11) prepared from PC-3 cells treated with thapsigargin (50 nM) and exposed to (**E**) 1% O_2_ or (**F**) DMOG (1 mM) as indicated. All values are normalised to 18S rRNA and fold change calculated from control samples prepared from untreated cells. Significance was calculated relative to untreated control using a one-way ANOVA with Dunnett's multiple comparisons test.

Levels and activation of HIF can be altered by transcriptional control of HIF subunits (2). However, steady state levels of HIF1α mRNA are unaltered by treatment with thapsigargin, hypoxia or hypoxia mimetics as indicated by qRT-PCR analysis of HIF1α mRNA levels (Figure 3A, B, Supplemental Figure S4A–E). HIF1α mRNA associated with active ribosomes was measured to determine the rate of HIF1α mRNA translation. Cellular protein synthesis, as well as translation rates of individual mRNAs, can be measured by polysome profiling; a technique in which free ribosomes can be separated from mRNA bound ribosomes (polysomes) on a sucrose gradient. Hypoxia results in a moderate decrease in the translation rates in PC-3 cells as indicated by the increase in the number of monosomes detected in cells exposed to 1% O_2_ (Figure 3C). The decrease in global translation rates is independent of HIF activity, as the hypoxia mimetic, DMOG, has no significant effect on protein synthesis (Figure 3D). Surprisingly, activation of the UPR using thapsigargin did not significantly alter the global translation rates as measured by the number of actively translating ribosomes (polysomes) in either hypoxic or DMOG treated cells (Figure 3C and D, Supplemental Figure S5). To investigate the levels of individual mRNAs associated with actively translating ribosomes, cDNAs were prepared from fractions containing polysomes (Fractions 8–11) (Figure 3C, D and Supplemental Figure S3). Quantitative RT-PCR analysis of polysome-associated HIF1α mRNA revealed a reduction of actively translating HIF1α in thapsigargin treated samples in both hypoxic and DMOG treated cells (Figure 3E and F). These data suggest that HIF1α mRNA translation is sensitive to activation of the UPR.

### UPR-dependent suppression of HIF activity requires PERK

**Figure 4.**
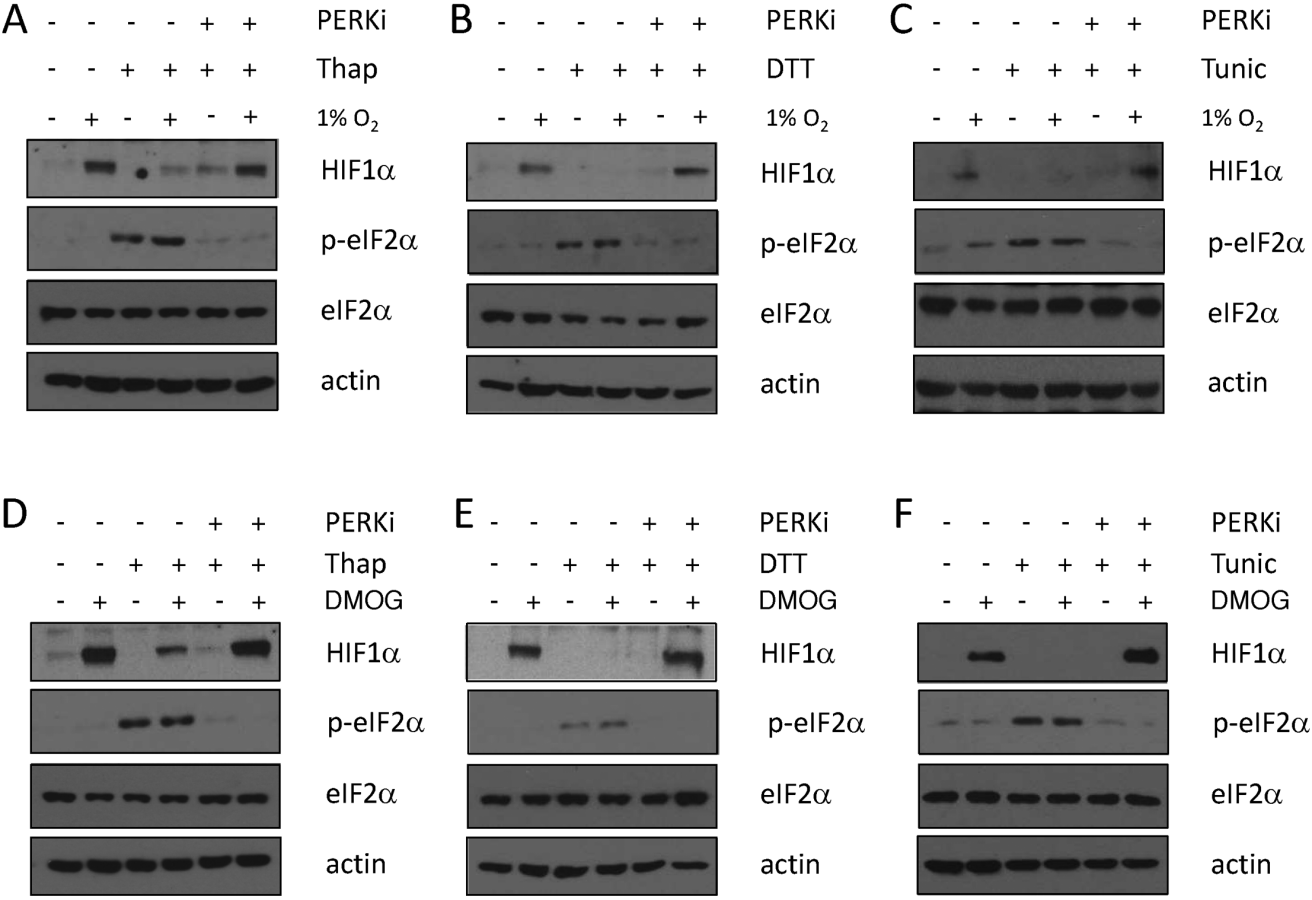
The UPR suppresses hypoxia-dependent HIF1α stabilisation in a PERK-dependent manner. PC-3 cells treated with (**A, D**) thapsigargin (50 nM) (**B, E**) DTT (2 mM) (**C, F**) 2.5 μg/ml tunicamycin and exposed to either (A–C) 1% O_2_, or (D–F) 1 mM DMOG for 3 h, were treated with the PERK inhibitor, GSK2606414 (0.3 μM) as indicated. WCLs were resolved by SDS PAGE and analysed by immunoblot using the indicated antibodies.

**Figure 5.**
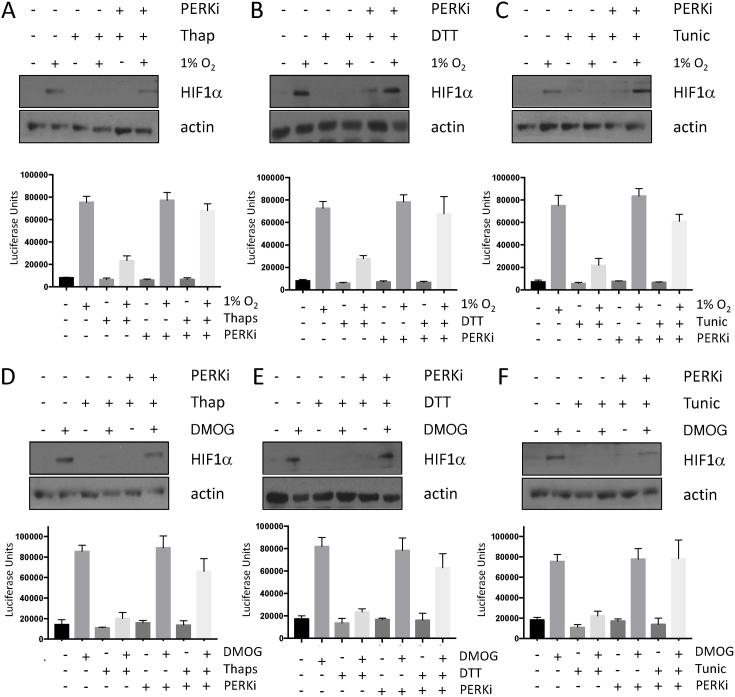
Inhibition of PERK rescues UPR-dependent suppression of HIF transcriptional activity. WCLs were prepared and luciferase activity was measured from U2OS cells stably expressing HRE-luciferase treated with (**A, D**) 50 nM thapsigargin, (**B, E**), 2 mM DTT or (C, F) 2.5 μg/ml tunicamycin and subsequently exposed to 1% O_2_ (A–C) 1 mM DMOG (D–F) for 7 h in the presence of the PERK inhibitor GSK2606414 (0.3 μM) as indicated. WCLs were resolved by SDS PAGE and analysed by immunoblot using the indicated antibodies. HRE luciferase results represent the mean plus S.D. of three independent experiments.

eIF2α is phosphorylated on S51 by four distinct kinases; PERK, heme-regulated inhibitor (HRI), protein kinase R (PKR), and general control non-depressible 2 (GCN2) which are activated by ER-stress, heme depletion, viral infection and amino acid starvation, respectively (30). Inhibition of PERK using GSK 2606414, a small molecule inhibitor, prevented thapsigargin-induced eIF2α phosphorylation consistent with the induction of ER stress (Figure 4A). Immunoblot analysis demonstrated that PERK inhibition reversed thapsigargin-dependent reduction of HIF1α levels (Figure 4A). Inhibition of PERK rescued the HIF1α defect from both hypoxic and DMOG treated cells exposed to DTT and tunicamycin (Figure 4B–F). PERK inhibition alone did not increase HIF1α levels in hypoxic cells, suggesting it is acting to reverse inhibition, rather than as a direct activator (Supplemental Figure S6). Inhibition of PERK was also sufficient to restore HIF1α levels in U2OS HRE-Luc cells exposed to UPR agonists (Figure 5A–F). Treatment with PERK inhibitor alone was not sufficient to elevate HIF1α levels or activity in the absence of UPR agonists (Supplemental Figure S6). Importantly, treatment with UPR agonists, either alone or in combination with GSK2606414 did not significantly alter levels of TNF induced NF-κB activity; indicating that UPR-dependent changes in HIF activity are specific (Supplemental Figure S7A–D). Together these results demonstrate that activation of the UPR inhibits HIF signaling in a PERK-dependent manner.

### UPR-dependent suppression of HIF-target genes is reversed by PERK inhibition

**Figure 6.**
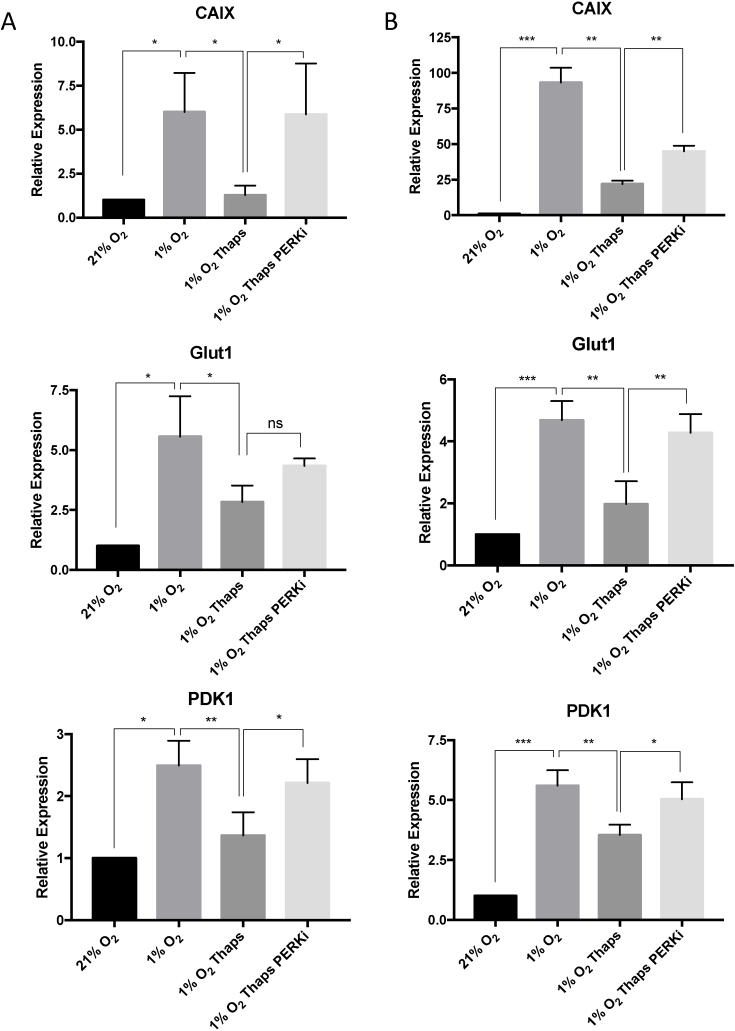
Inhibition of PERK rescues UPR-dependent suppression of HIF-target genes. (**A**) Quantitative RT–PCR analysis of CAIX, Glut1 and PDK1 mRNA prepared from PC-3 cells treated with 50 nM thapsigargin and 0.3 μM GSK2606414 as indicated, and exposed to 1% O_2_ for 7 h. All values are normalised to RPL13A mRNA and fold change calculated from control samples prepared in normoxic conditions. (**B**). Quantitative RT–PCR analysis of CAIX, Glut1 and PDK1 mRNA prepared from U2OS cells treated with 50 nM thapsigargin and 0.3 μM GSK2606414 as indicated and exposed to 1% O_2_ for 7 h. All values are normalised to RPL13A mRNA and fold change calculated from control samples prepared in normoxic conditions. Significance was calculated relative to hypoxic controls using a one-way ANOVA with Dunnett's multiple comparisons test.

To examine the role of the UPR on the expression of hypoxia-responsive genes, we performed quantitative real time PCR analysis of the expression of HIF1 target genes in PC-3 and U2OS cells treated with thapsigargin. PC-3 and U2OS cells were treated with thapsigargin and the PERK inhibitor and subjected to hypoxia for 7 h (Figure 6A and B). RNA was isolated from these cells and was used to quantitate the transcript levels of the canonical HIF1 target genes; CAIX, GLUT1 and PDK1. As expected, exposure to low oxygen resulted in an increase in expression of all of these genes, as compared to control (Figure 6A and B). Expression of HIF target genes is suppressed in hypoxic cells treated with thapsigargin and inhibition of PERK was sufficient to reverse the UPR-dependent suppression of HIF-responsive genes in both PC-3 and U2OS cells (Figure 6A and B).

### PERK Inhibition rescues HIF1α translation in thapsigargin treated cells

**Figure 7.**
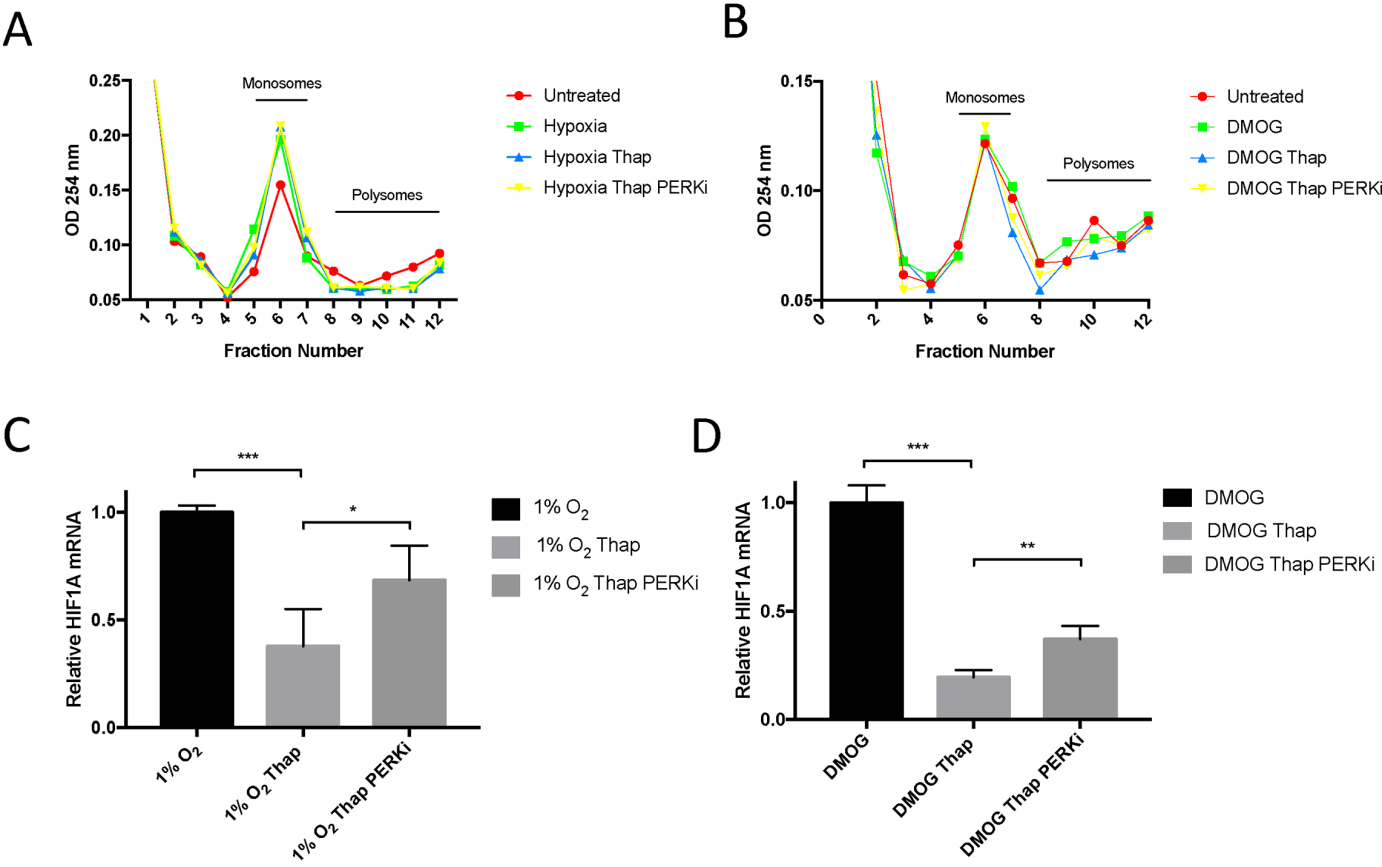
UPR-dependent suppression of HIF1α translation in attenuated by PERK. Representative polysome profiles of PC-3 cells treated with 50 nM thapsigargin and 0.3 μM GSK2606414 and exposed to (**A**) 1% O_2_ or (**B**) 1 mM DMOG for 7 h. Fractions containing monosomes and polysomes are indicated on the graph. Quantitative RT-PCR analysis of HIF1α mRNA prepared from polysomal fractions (8–11) prepared from PC-3 cells treated with 50 nM thapsigargin and 0.3 μM GSK2606414 then exposed to (**C**) 1% O_2_ or (**D**) 1 mM DMOG for 7 h as indicated. All values are normalised to 18S rRNA and fold change calculated from control samples prepared from hypoxic or DMOG treated cells. Significance was calculated relative to untreated control using a one-way ANOVA with Dunnett's multiple comparisons test.

Polysomal profiling was performed on PC-3 cells treated with thapsigargin and GSK2606414 to measure actively translating ribosomes. Surprisingly, PERK inhibition did not have an obvious effect on global protein synthesis, as measured by the number of actively translating ribosomes in hypoxic or DMOG treated cells (Figure 7A and B). Treatment with GSK2606414 did however significantly reverse the thapsigargin-dependent suppression of HIF1α mRNA translation in both hypoxic and DMOG treated PC-3, indicating that the UPR-dependent decrease in HIF1α translation is in part dependent on PERK activity (Figure 7C and D).

### HIF1α mRNA/ YB-1 interaction is disrupted in thapsigargin treated cells

**Figure 8.**
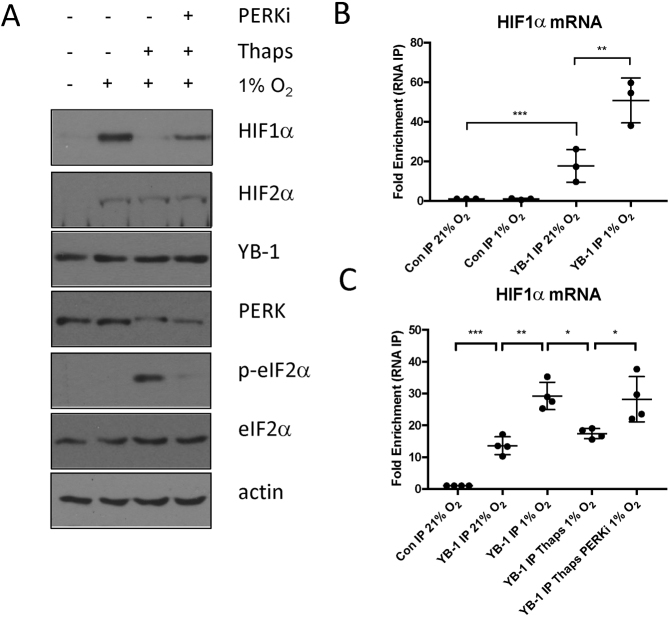
Activation of the UPR suppresses HIF1α translation by preventing YB-1 binding to HIF1α mRNA. (**A**) WCLs were prepared from PC-3 cells treated with thapsigargin, 0.3 μM GSK2606414 and exposed to 1% O_2_ as indicated. WCLs were resolved by SDS PAGE and analysed by immunoblot using the indicated antibodies. (**B**) HIF1α mRNA bound to YB-1 as measured by qRT-PCR following YB-1 immunoprecipitation. (**C**) HIF1α mRNA bound to YB-1 in the presence of 1% O_2_, 50 nM thapsigargin and 0.3 μM GSK2606414 as indicated. RNA IP values are normalised against inputs and presented as means ± SD. Significance was calculated relative to untreated control using a one-way ANOVA using the Dunnett multiple comparison test.

Maintaining high levels of HIF1α mRNA translation is critical for full activation of HIF during prolonged hypoxia (11,12). Several regulatory factors have been identified that enhance or reduce HIF1α translation by directly binding to HIF1α mRNA. One such factor is the Y-box binding protein 1 (YB-1) that binds directly to the 5′ UTR of HIF1α mRNA to sustain high levels of HIF1α in hypoxia (31). Previous studies have reported YB-1 activity and subcellular localisation is sensitive to conditions of ER-stress. YB-1, normally diffusely present in the cytosol, relocalises to ribonucleoprotein complexes known as stress granules in thapsigargin treated cells (32). We therefore examined whether YB-1 played a role in UPR-dependent reduction in HIF1α translation. As the total levels of YB-1 are not altered by either hypoxia or activation of the UPR (Figure 8A), we investigated if the YB-1/ HIF1α mRNA was disrupted by thapsigargin treatment. YB-1 was efficiently precipitated from cell extracts using a specific polyclonal antibody (Supplemental Figure S8A). cDNA prepared from YB-1 precipitates and analysed by quantitative RT-PCR revealed an increased association between YB-1 and the HIF1α transcript, consistent with its role in maintaining HIF1α translation in low oxygen (Figure 8B). In the presence of the UPR agonist, thapsigargin, the levels of YB-1 associated with the HIF1α transcript are reduced (Figure 8C). The reduction in the YB-1 / HIF1α mRNA interaction is consistent with the reduction in HIF1α translation. YB-1 interaction with HIF1α mRNA was specific as no significant binding of the IL-8 transcript to YB-1 was observed (Supplemental Figure S8B). Thapsigargin-dependent reduction of the YB-1/ HIF1α mRNA interaction was partially reversed by GSK2606414, consistent with the increase in HIF1α translation (Figure 8C). Our data indicate that activation of the UPR reduces the interaction between the translational activator YB-1 and the HIF1α mRNA in a PERK-dependent manner.

### Thapsigargin sensitises cells to hypoxic stress

**Figure 9.**
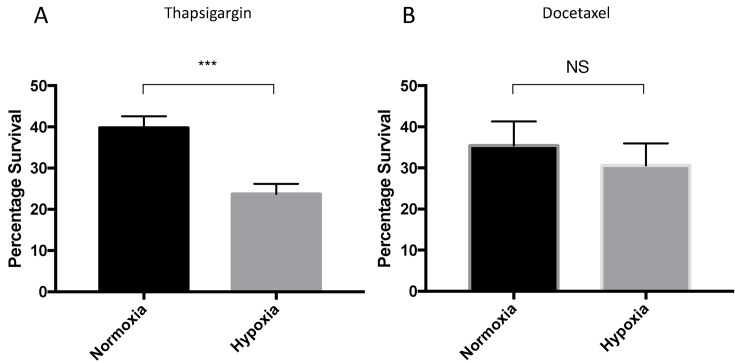
Activation of the UPR sensitises cells to hypoxic stress. (**A**) PC-3 cells were treated with 100nM thapsigargin and incubated for 24 h in 1% or 21% O2. Cell viability was measured by prestoblue assay and values normalised to untreated controls. (**B**) As in (A) with 100 nM Docetaxel. Significance was calculated relative to untreated control using an unpaired *t*-test.

Activation of the HIF family of transcription factors is a critical component of the cellular response to low oxygen. In solid tumor cells elevated levels of HIF1α contribute to the malignant phenotype by promoting the expression of pro-angiogenic and pro-survival gene products. As thapsigargin decreases HIF1α levels and activity we examined if prostate cancer cell lines were more sensitive to thapsigargin in hypoxic cells. PC-3 cells were treated with thapsigargin and incubated at normoxia and hypoxia for 24 h. The viability of hypoxic PC-3 cells was significantly reduced by thapsigargin treatment, consistent with the reduction of HIF1α activity (Figure 9A). Increased sensitivity to thapsigargin in low oxygen conditions was specific, as normoxic and hypoxic PC-3 cells were equally sensitive to the antimitotic agent, docetaxel (Figure 9B). The data suggest that activation of the UPR reduces the HIF-dependent hypoxic response by impairing HIF1α mRNA translation.

## DISCUSSION

Clinical and experimental data suggest that oxygen homeostasis and HIF activity is disrupted in multiple human pathologies such as heart disease, cancer, cardio vascular disease and chronic obstructive pulmonary disease (1,33). The majority of studies examining how HIF is deregulated have focused on protein stability, however control of HIF1α translation remains relatively understudied despite its major contribution to the HIF-dependent hypoxic response. The results from the present study indicate that activation of the UPR during moderate hypoxia can attenuate the HIF-dependent transcriptional program. UPR agonists prevent the full activation of HIF by inhibiting HIF1α mRNA translation, resulting in a decrease in HIF1 transcriptional activity and a suppression of HIF1-target gene expression in a variety of cell lines.

The UPR is a major determinant of cell survival in response to various conditions of cellular stress and is associated with various human pathologies including inflammatory diseases, diabetes and cancer (22). The UPR is characterised by the activation of three parallel signalling pathways: PERK-dependent phosphorylation of eIF2α, inositol-requiring protein 1α (IRE1α)–X-box binding protein 1 (XBP1) and activating transcription factor 6α (ATF6α). Our data indicate that activation of the PERK pathway suppresses the full activation of HIF target gene expression. Polysome profiling suggests that HIF1α mRNA translation is extremely sensitive to UPR activation. These data were unexpected, as HIF1α is efficiently stabilised in severely hypoxic cells in which the UPR is activated (23). Our results indicate that activation of the UPR does not promote HIF1α accumulation, but severely suppresses it, indicating that alternative signaling pathways must be activated to maintain HIF1α biogenesis in severely hypoxic cells. Interestingly, our data show that hypoxia-dependent accumulation of HIF2α is unaffected by UPR activation, suggesting the translational control of HIF1α mRNA by YB-1 is specific for the HIF1α subunit (Figure 8A).

Accumulating evidence indicates HIF1α translation is an important mechanism to control HIF activity in cells (11,12). HIF1α mRNA has non-coding regions at both the 5′ and 3′ ends of the transcript that can be bound by regulatory proteins to control rates of HIF1α biogenesis (31,34). However, the signaling pathways that regulate their binding to the HIF1α transcript remain poorly defined (31,34). YB-1 is a multifunctional nucleic acid-binding protein that can directly bind to and activate translation of HIF1α mRNA to promote sarcoma cell invasion and enhanced metastatic capacity in vivo (31). In our present study we find that activation of the UPR under conditions of hypoxic stress reduces the interaction of YB1 with HIF1α mRNA, reducing HIF1α mRNA translation and causes a decrease in HIF1α levels. Total YB-1 is not altered by activation of the UPR or hypoxic stress, however when cells experience ER stress YB-1 alters its subcellular distribution to localise to stress granules; discrete riboprotein complexes in the cytoplasm of cells (32). This rapid redistribution of YB-1 results in less YB-1 being associated with HIF1α mRNA, suppressing its translation.

In addition to its role in ER homeostasis, the UPR has emerged as a key mediator of DNA replication, energy metabolism and cellular activation of apoptosis (35,36). UPR components are often deregulated in malignant cells and the activation of the UPR is thought to contribute to tumor development (37). However, whether activation of the UPR is positive or negative for tumor progression remains unclear (38,39). UPR agonists such as thapsigargin and tunicamycin have been shown to be effective in promoting tumor cell death (40,41). Indeed, analogues of thapsigargin are currently in clinical trials as prostate cancer therapeutics (42). The effectiveness of these agents in targeting tumor cells may be due to their ability to target the HIF pathway. Indeed, histological analysis of implanted tumors in nude mice treated with tunicamycin revealed reduced growth, vasculature and VEGF levels, classical signs of reduced HIF activity (43). Our data suggest that hypoxic tumor cells display increased sensitivity to UPR agonists, indicating that activation of the UPR may be a strategy to target hypoxic, solid tumors.

Collectively our data reveal that ER-stress regulates the interaction between the YB-1 protein and HIF1α mRNA, which can alter rates of HIF1α protein synthesis. UPR-dependent suppression of HIF1α may provide a novel strategy for targeting aberrant HIF activity in human disease.

## Supplementary Material

Supplementary Data
