# Supplementary material for: PERK/eIF2α signaling inhibits HIF-induced gene expression during the unfolded protein response via YB1-dependent regulation of HIF1α translation
Source: Nucleic Acids Res. 2018 Feb 26;46(8):3878–90. doi: 10.1093/nar/gky127 (PMC5934640; doi:10.1093/nar/gky127)
Supplement: Supplementary Data [file gky127_supp.zip › nar-03322-v-2017 -File011.docx]

**Supplemental Figure 1 Modulation of cytoplasmic calcium levels does not interfere with HIF1α accumulation.** (A) PC-3 cells were treated ionomycin as indicated and exposed to 1% O_2_ for 2h. Whole-cell lysates (WCLs) prepared from these cells were subjected to immunoblot analysis to assess expression levels of the indicated proteins. (B) Lysates prepared from PC-3 cells treated with 50nM thapsigargin, 2.5uM ionomycin and exposed to 1% O_2_ for 2 hours were immunoblotted using the indicated antibodies (C) PC-3 cells were treated EGTA as indicated and exposed to 1% O_2_ for 2h. Whole-cell lysates (WCLs) prepared from these cells were subjected to immunoblot analysis to assess expression levels of the indicated proteins. (D) Lysates prepared from PC-3 cells treated with 50nM thapsigargin, and 1μM EGTA, and exposed to 1% O_2_ for 2 hours as indicated were subject to immunoblot analysis.

**Supplemental Figure 2 Activation of the UPR reduces the accumulation of HIF1α in response to CoCl_2_** PC-3 cells treated with 50nM thapsigargin and rated with 500μM CoCl_2_ as indicated. WCLs prepared from these cells were subjected to immunoblot analysis to assess expression levels of the indicated proteins.

**Supplemental Figure 3 Thapsigargin does not alter HIF1α protein stability** PC-3 cells treated with 50nM thapsigargin, the proteasome inhibitor MG132 (10μM) and exposed to (A) 1% O_2_ or (B) 1mM DMOG. PC-3 cells treated with 50nM thapsigargin and the proteasome inhibitors (C) 10μM MG132 or (D) 10μM lactacystin (lact). PC-3 cells treated with thapsigargin and incubated in 1% O_2_ for 3 hours were co-treated with the lysosomal inhibitors (E) 200nM baflomycin A (BAF-A) and (F) 10μM chloroquine (CQ). Whole-cell lysates (WCLs) prepared from these cells were subjected to immunoblot analysis to assess expression levels of the indicated proteins.

**Supplemental Figure 4** Quantitative RT–PCR analysis of HIF1α mRNA prepared from **(A)** PC-3 **(B)** U2OS **(C)** MCF7 or **(D)** COV-434 cells exposed to 1% O_2_ (hypox) or treated with 1mM DMOG for 7 hours. (E) Quantitative RT–PCR analysis of HIF1α mRNA from PC-3 cells treated with 50nM thapsigargin and exposed to 1% O_2_ for 7h. All values are normalized to RPL13A mRNA and fold change calculated from normoxic controls.

**Supplemental Figure 5 Thapsigargin does not alter total protein synthesis in PC-3 cells** RNA purified from polysomal fractions prepared from PC-3 cells treated with DMOG (1mM) and thapsigargin (50nM) collected from top to bottom of the sucrose density gradient visualised by agarose gel electrophoresis.

**Supplemental Figure 6 The UPR suppresses hypoxia-dependent HIF1α stabilization in a PERK-dependent manner**. PC-3 cells treated with Thapsigargin (50nM) and exposed to 1% O_2_ were treated with the PERK inhibitor, GSK2606414 (0.3μM) as indicated. WCLs were resolved by SDS PAGE and analyzed by immunoblot using the indicated antibodies.

**Supplemental Figure 7 Activating the UPR suppresses HIF activity in a PERK-dependent manner**. (A) Luciferase activity was measured in lysates prepared from U2OS cells stably expressing HRE-luciferase treated with 50nM thapsigargin, and the PERK inhibitor GSK2606414 (0.3μM) as indicated. HRE luciferase results represent the mean plus S.D. of three independent experiments. (B-D) Luciferase activity was measured in lysates prepared from U2OS cells stably expressing NF-κB-luciferase reporter treated with (B) 50nM thapsigargin, (C) 2mM DTT, (D) 2.5μg/ml tunicamycin and the PERK inhibitor GSK2606414 (0.3μM) in the presence or absence of 10ng/ml TNF as indicated. HRE luciferase results represent the mean plus S.D. of three independent experiments.

**Supplementary Figure 8 Activation of the UPR does not impair TNF-induced NF-κB activity**  **(A)** YB-1 was immunopreciptiated from PC-3 cells treated with 1% O_2_ and 50nM thapsigargin as indicated. The YB-1 precipitates were immunoblotted with a YB-1 antibody **(B)** IL-8 mRNA bound to YB-1 as measured by qRT-PCR following YB-1 immunoprecipitation.
