# Supplementary material for: PERK/eIF2α signaling inhibits HIF-induced gene expression during the unfolded protein response via YB1-dependent regulation of HIF1α translation
Source: Nucleic Acids Res. 2018 Feb 26;46(8):3878–90. doi: 10.1093/nar/gky127 (PMC5934640; doi:10.1093/nar/gky127)
Supplement: Supplementary Data [file gky127_supp.zip › nar-03322-v-2017 -File012.pptx]

## Slide 1
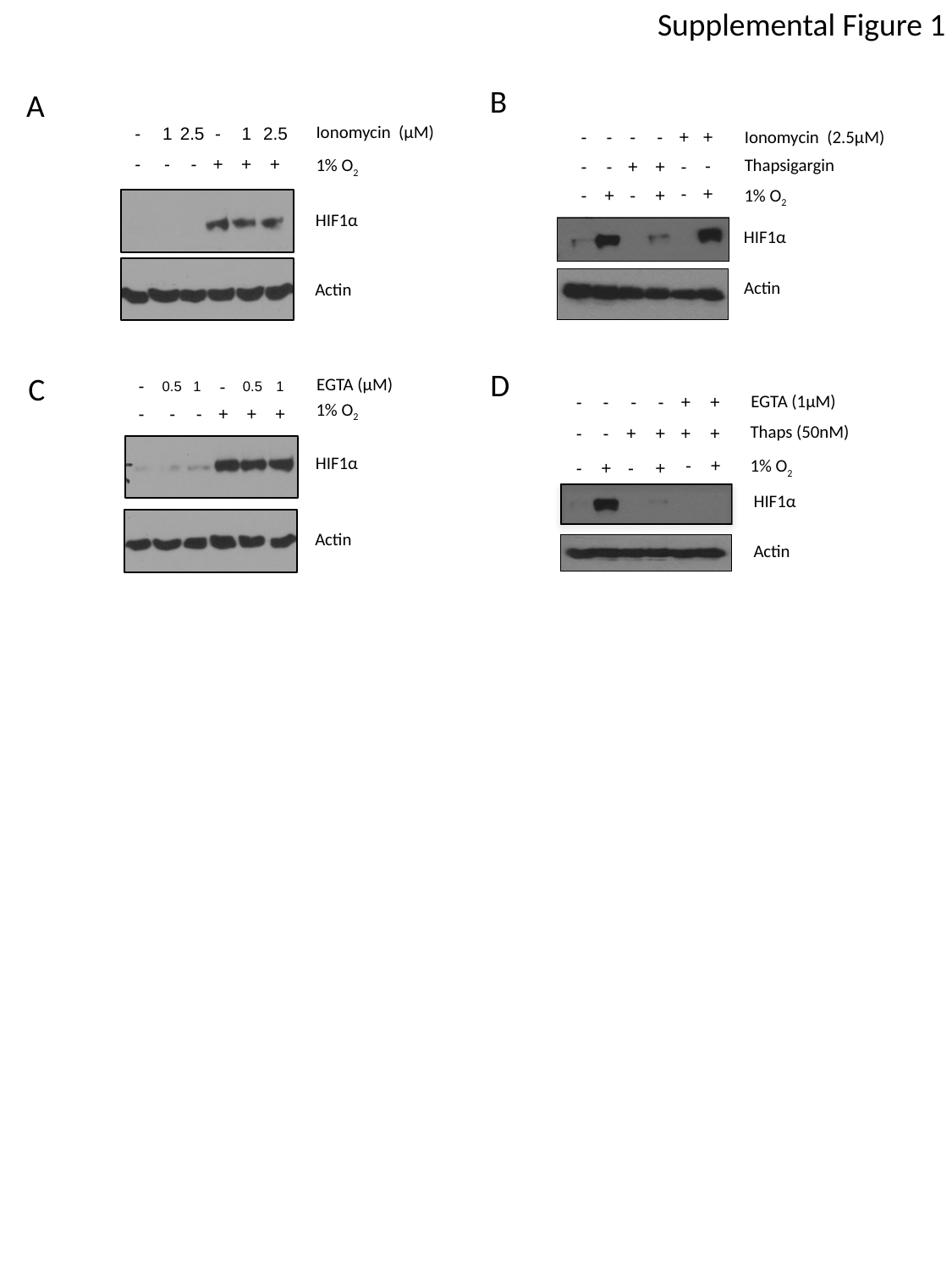

Supplemental Figure 1
B
A
Ionomycin (µM)
-
1
2.5
-
1
2.5
-
-
-
-
+
+
Ionomycin (2.5µM)
+
-
-
-
+
+
Thapsigargin
1% O2
-
-
-
+
+
-
-
+
-
+
-
+
1% O2
HIF1α
HIF1α
Actin
Actin
D
C
EGTA (µM)
-
-
0.5
1
0.5
1
EGTA (1µM)
-
-
-
-
+
+
1% O2
+
-
-
-
+
+
Thaps (50nM)
-
-
+
+
+
+
HIF1α
-
+
1% O2
-
+
-
+
HIF1α
Actin
Actin

## Slide 2
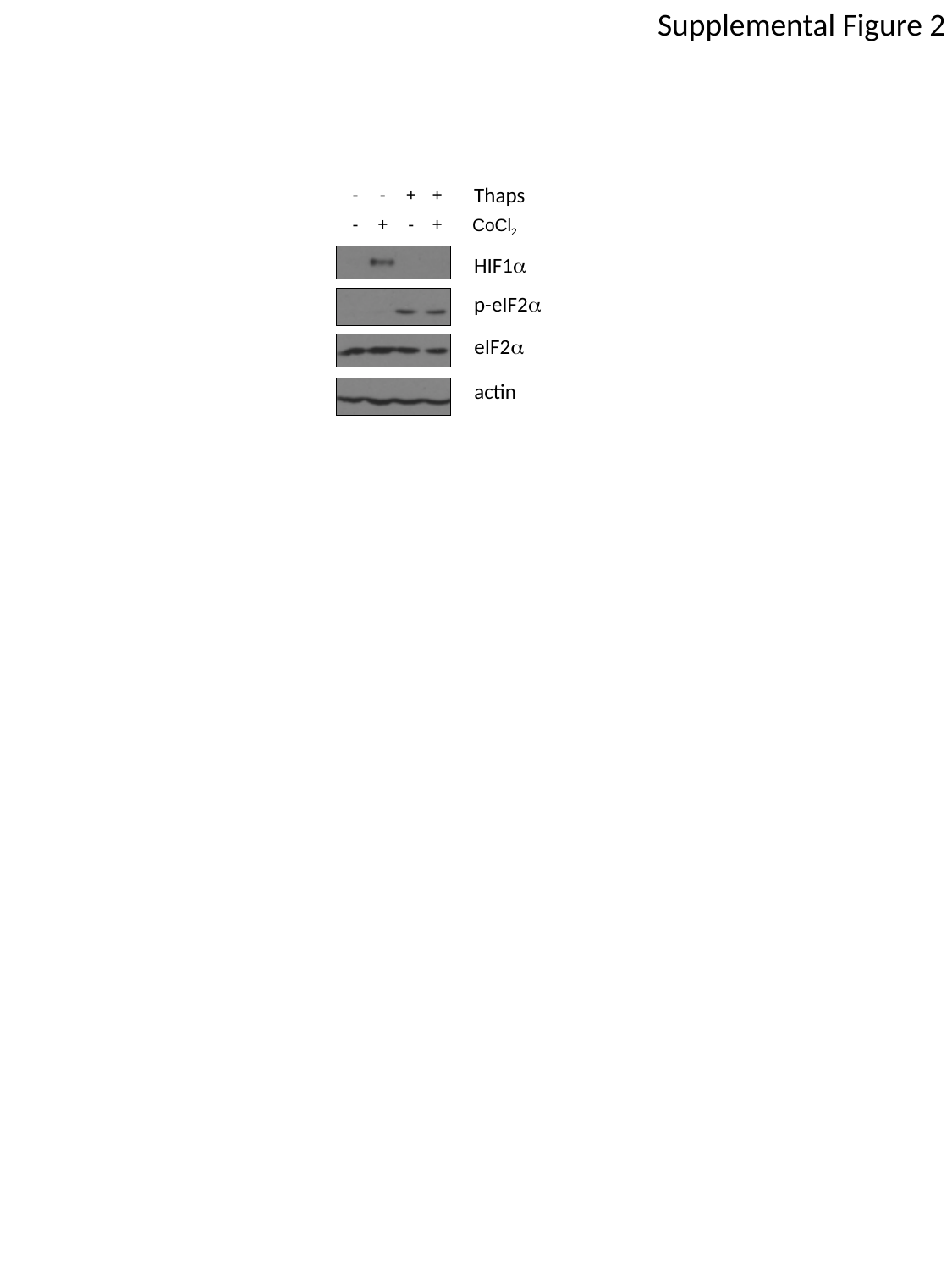

Supplemental Figure 2
Thaps
-
-
+
+
-
+
-
+
CoCl2
HIF1a
p-eIF2a
eIF2a
actin

## Slide 3
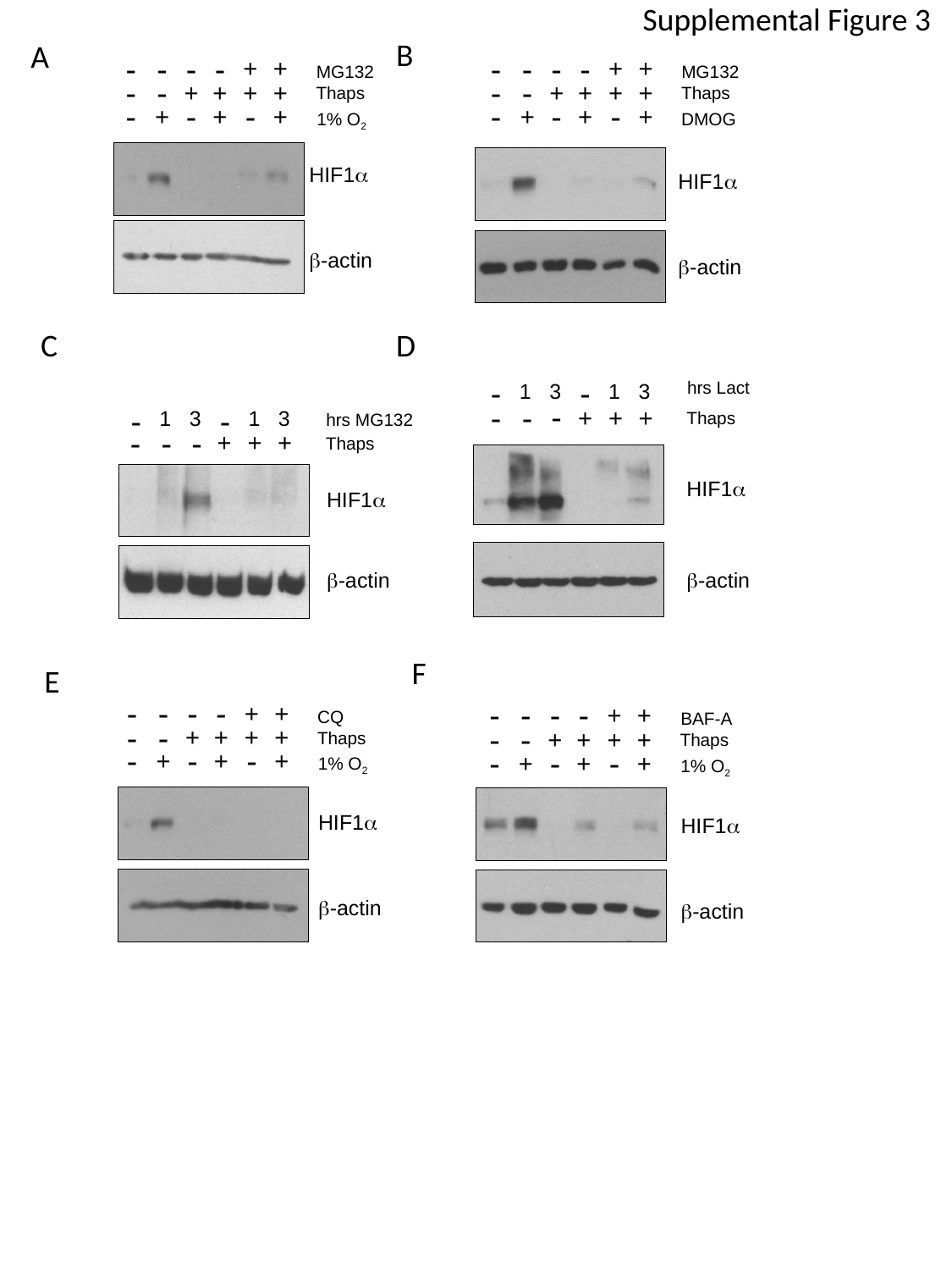

Supplemental Figure 3
B
A
-
-
-
-
+
+
MG132
-
-
+
+
+
+
Thaps
+
+
+
1% O2
-
-
-
-
-
-
-
+
+
MG132
-
-
+
+
+
+
Thaps
+
+
+
DMOG
-
-
-
HIF1a
HIF1a
b-actin
b-actin
C
D
-
-
hrs Lact
1
3
1
3
-
-
-
-
-
+
+
+
1
3
1
3
Thaps
hrs MG132
-
-
-
+
+
+
Thaps
HIF1a
HIF1a
b-actin
b-actin
F
E
-
-
-
-
+
+
CQ
-
-
+
+
+
+
Thaps
-
-
-
+
+
+
1% O2
-
-
-
-
+
+
BAF-A
-
-
+
+
+
+
Thaps
-
-
-
+
+
+
1% O2
HIF1a
HIF1a
b-actin
b-actin

## Slide 4
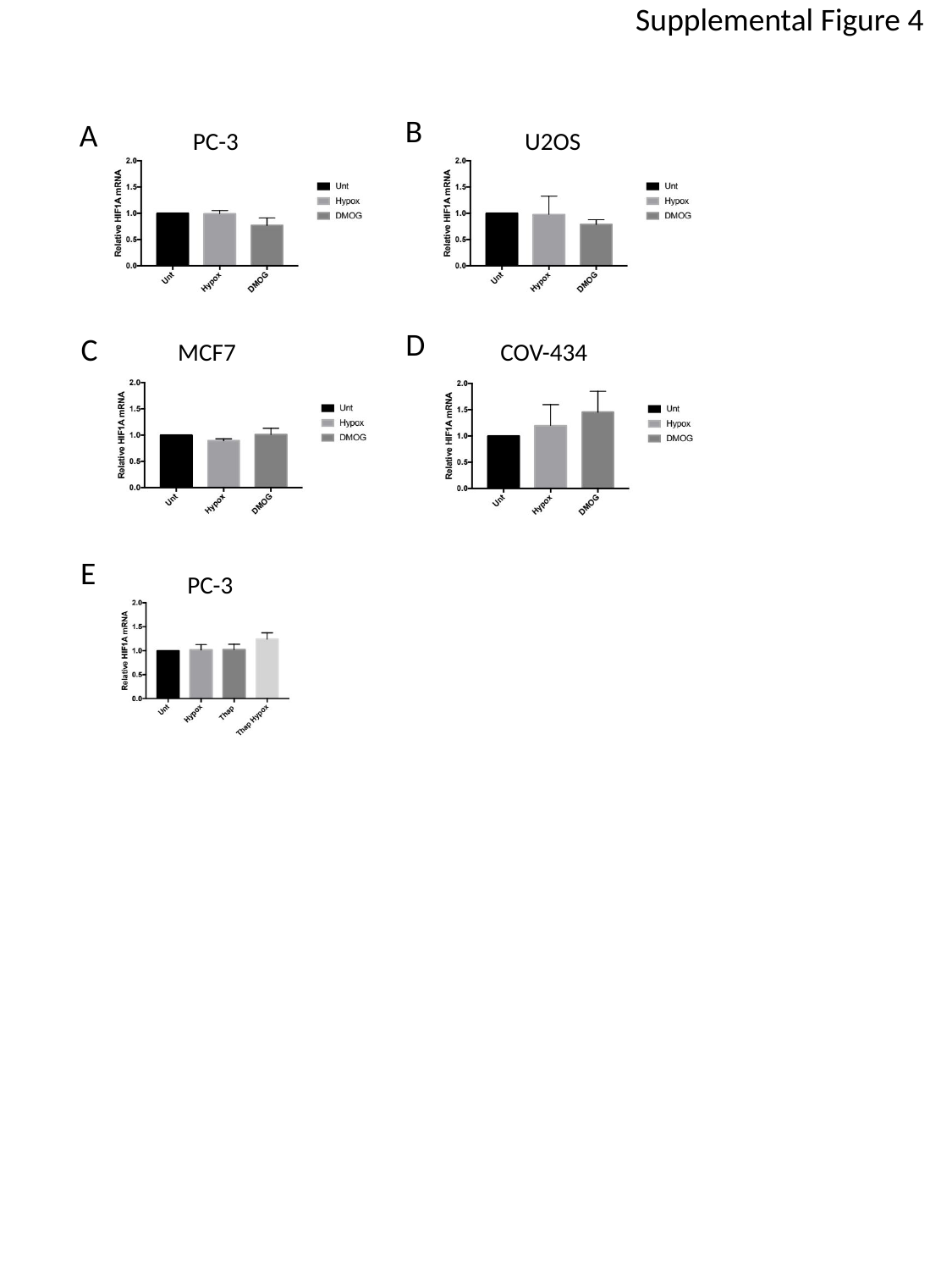

Supplemental Figure 4
B
A
PC-3
U2OS
D
C
MCF7
COV-434
E
PC-3

## Slide 5
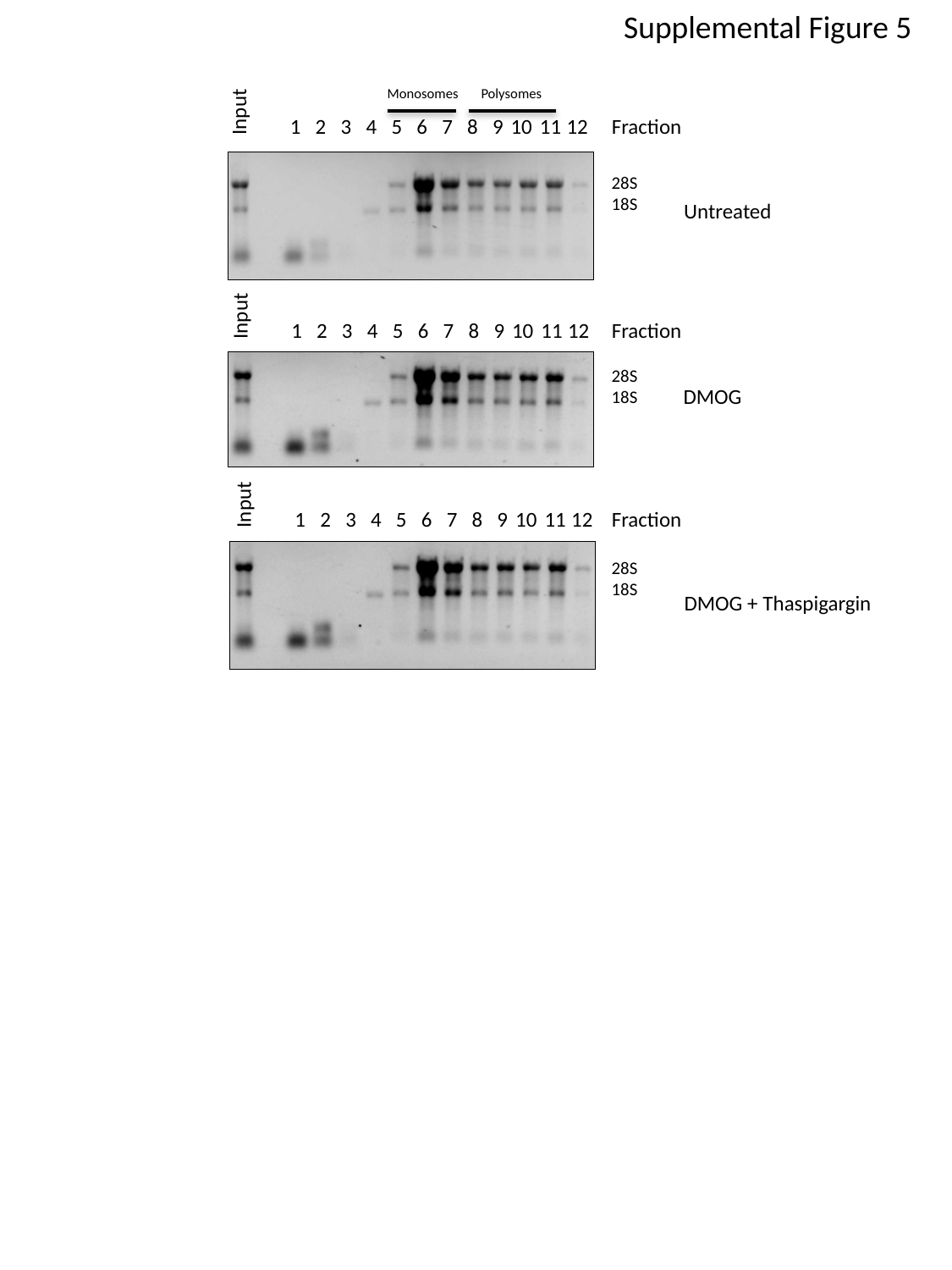

Supplemental Figure 5
Monosomes
Polysomes
Input
1
2
3
4
5
6
7
8
9
10
11
12
Fraction
28S
18S
Untreated
Input
1
2
3
4
5
6
7
8
9
10
11
12
Fraction
28S
DMOG
18S
Input
1
2
3
4
5
6
7
8
9
10
11
12
Fraction
28S
18S
DMOG + Thaspigargin

## Slide 6
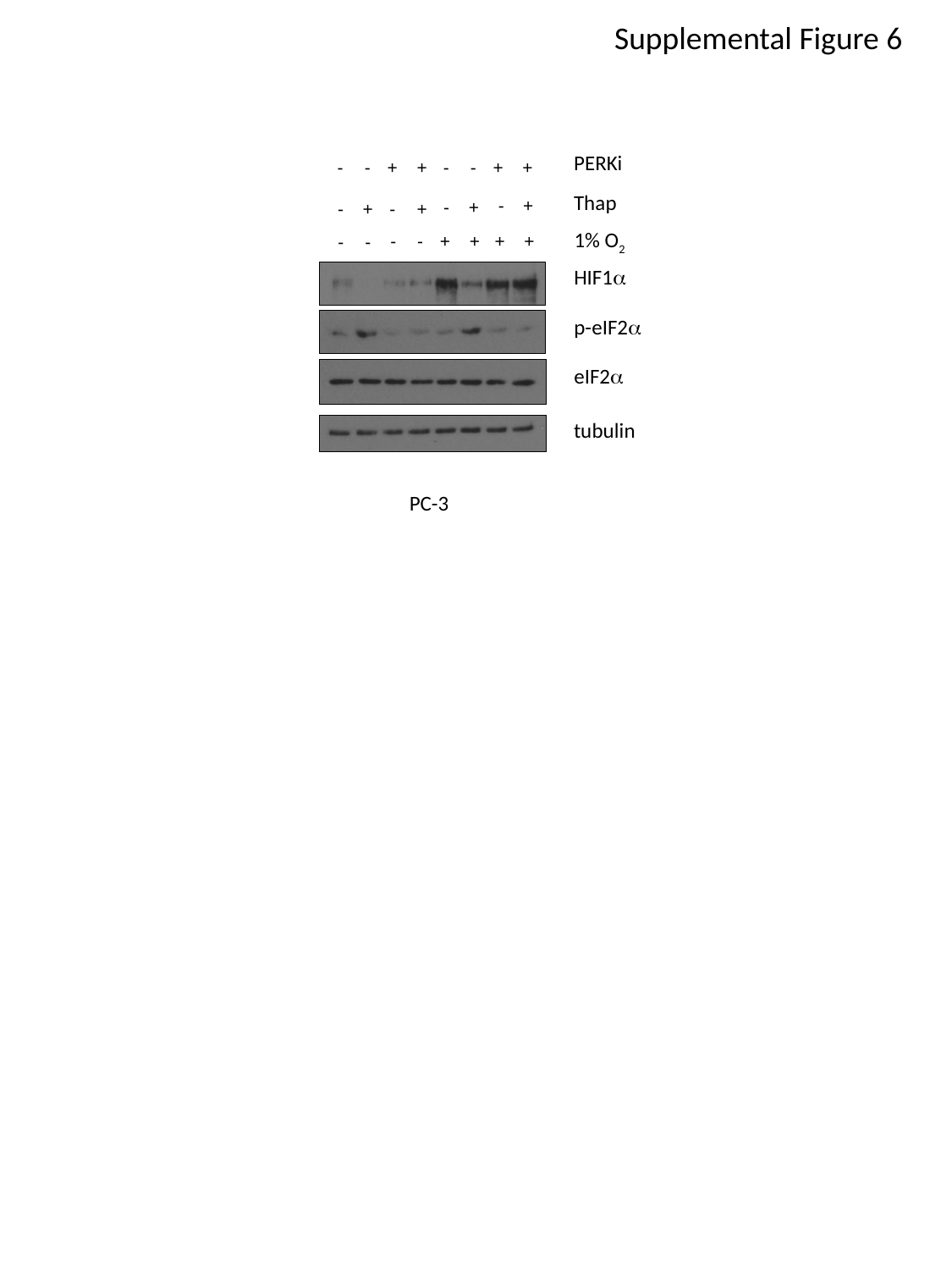

Supplemental Figure 6
PERKi
-
-
+
+
-
-
+
+
Thap
-
+
-
+
-
+
-
+
1% O2
-
-
+
+
+
+
-
-
HIF1a
p-eIF2a
eIF2a
tubulin
PC-3

## Slide 7
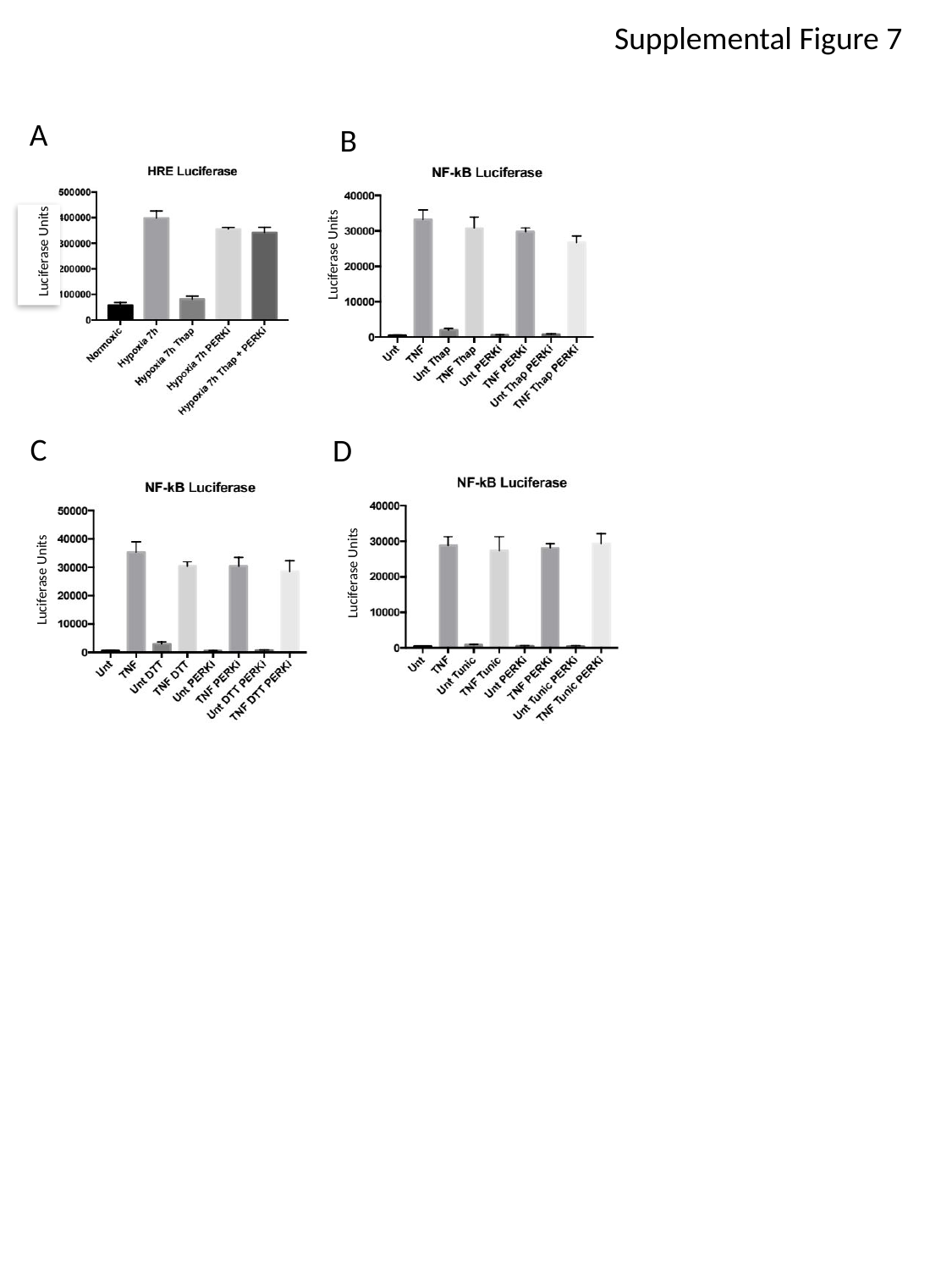

Supplemental Figure 7
A
B
Luciferase Units
Luciferase Units
C
D
Luciferase Units
Luciferase Units

## Slide 8
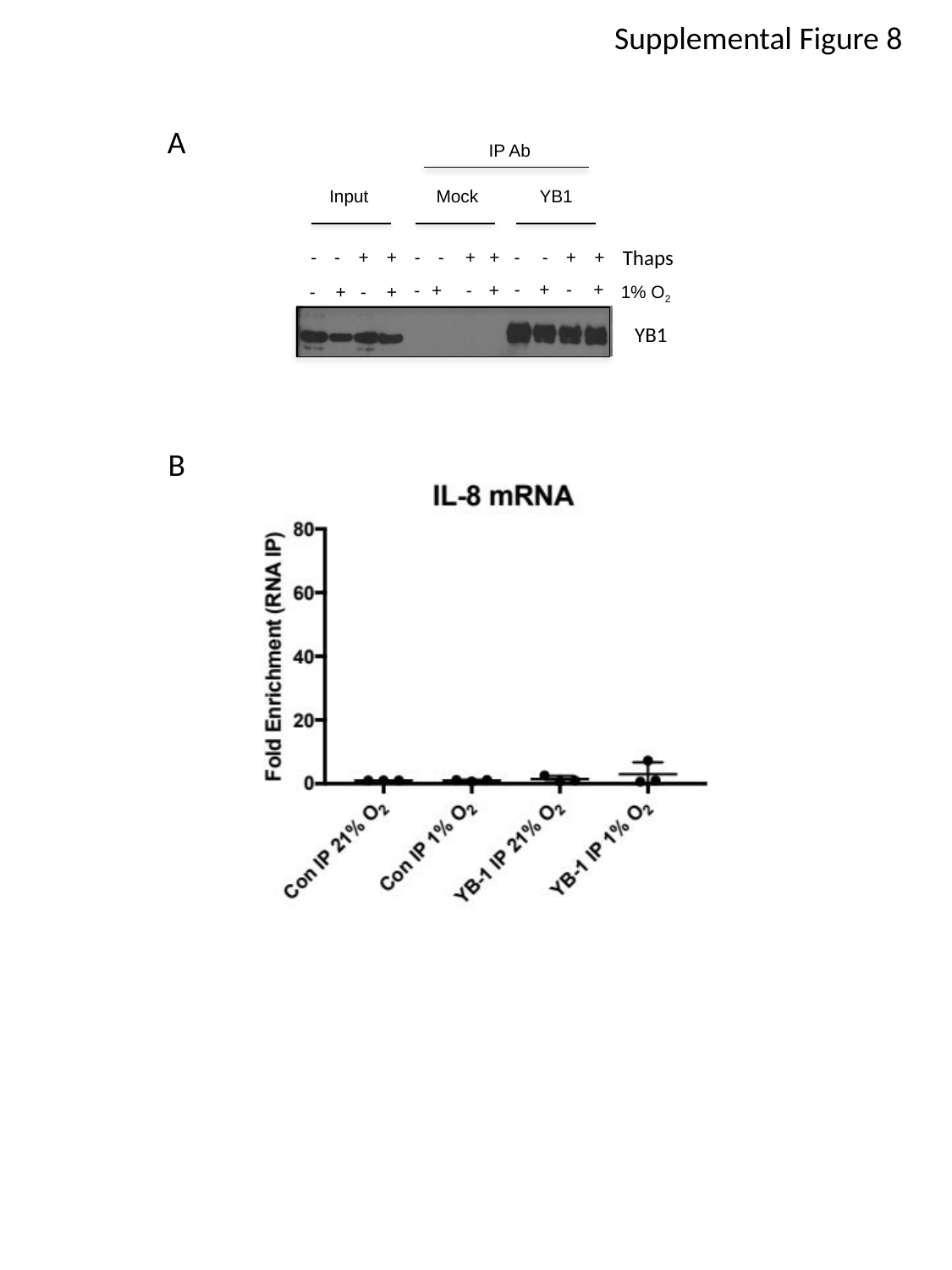

Supplemental Figure 8
A
IP Ab
Input
Mock
YB1
Thaps
-
-
+
+
-
-
+
+
-
-
+
+
-
+
-
+
-
+
-
+
-
+
-
+
1% O2
YB1
B
